# Wireless Sensor Networks for Long-Term Monitoring of Urban Noise

**DOI:** 10.3390/s18093161

**Published:** 2018-09-19

**Authors:** Courtney Peckens, Cédric Porter, Taylor Rink

**Affiliations:** Department of Engineering, Hope College, 27 Graves Place, Holland, MI 49422-9000, USA; cedric.porter@hope.edu (C.P.); taylor.rink@hope.edu (T.R.)

**Keywords:** noise monitoring, sensor node design, wireless sensor networks, long-term monitoring, smart city, acoustic sensing

## Abstract

Noise pollution in urban environments is becoming increasingly common and it has potential to negatively impact people’s health and decrease overall productivity. In order to alleviate these effects, it is important to better quantify noise patterns and levels through data collection and analysis. Wireless sensor networks offer a method for achieving this with a higher level of granularity than traditional handheld devices. In this study, a wireless sensing unit (WSU) was developed that possesses the same functionality as a handheld sound level meter. The WSU is comprised of a microcontroller unit that enables on-board computations, a wireless transceiver that uses Zigbee protocol for data transmission, and an external peripheral board that houses the microphone transducer. The WSU utilizes on-board data processing techniques to monitor noise by computing equivalent continuous sound levels, *L_eqT_*, which effectively minimizes data transmission and increases the overall longevity of the node. Strategies are also employed to ensure real-time functionality is maintained on the sensing unit, with a focus on preventing bottlenecks between data acquisition, data processing, and wireless transmission. Four units were deployed in two weeks field validation test and were shown to be capable of monitoring noise for extended periods of time.

## 1. Introduction

According to the United Nations, 55% of the world’s population resides in urban settings, and it is projected that this percentage will continue to increase up to 68% by 2050 [1]. This rapid and concentrated population growth creates many new challenges for urban planners and engineers as they seek to effectively manage resources while maintaining a high quality of living in these environments. To aid in this effort, the concept of smart cities, with an increased emphasis on urban monitoring and data gathering, has emerged in the last decade [2,3]. Most leading cities in Europe, the United States, and Asia have started trending toward such initiatives by adopting some form of ICT (Information and Communication Technologies) to aid in urban management [4]. However, full scale deployments of smart cities remain in preliminary stages because of challenges that are associated with the necessary development and implementation of governance structure, appropriate partnership formations between the private sector and the government, and effective ICT infrastructure integration [5]. In particular, the ICT infrastructure integration has been the focus of numerous researchers who seek to offer appropriate solutions for data acquisition and management for various problem areas in urban settings.

Rapid urban growth has led to an increase in noise pollution, which has been identified as one of the most major health concerns in these settings and is typically generated by road traffic, railways, airports, and industry [6]. High levels of environmental noise have been shown to have negative health impacts on humans and can lead to increases in blood pressure, cardiovascular disease, depression, nervousness, anxiety, and mood swings [7,8], as well as a decrease in overall productivity [9]. Furthermore, it is recommended by the World Health Organization that long-term exposure is limited to 55 dB to prevent elevated blood pressure and to less than 40 dB during the night to prevent sleep disturbance and awakenings [10]. Urban areas operate well above this range with noise levels typically peaking around 80 dB and nighttime sound levels typically ranging between 50 and 55 dB [11].

Europe has taken an active stance on minimizing noise pollution, and it has mandated the development of urban noise maps through the European Noise Directive 2002/49/EC (END) [12] in order to better understand patterns of noise. Further specifications for such noise maps have also been defined by the consequent Common Noise Assessment Methods (CNOSSOS-EU) [13]. Furthermore, the European Commission has dedicated significant funding toward projects related to noise, such as X-NOISE [14], which seeks to lower the exposure of the community to aircraft noise, and FONOMOC [15], which was established to aid in the exchange of knowledge and experience on noise monitoring systems. In the United States, on the other hand, it is recognized by the Environmental Protection Agency that noise pollution is a significant concern but the issue is left to be handled at the state and local level [16], and little funding has been dedicated toward research in this area. Efforts have persisted at the local level, however, with some cities deploying their own monitoring systems, such as the Array of Things (AoT) in Chicago, Illinois, USA, which includes a noise sensor. However, most noise collection in the United States is completed on an ad-hoc and sparsely populated basis. 

This paper seeks to add to the literature by demonstrating a long-term and low-cost noise monitoring platform that will aid in better understanding patterns and levels in urban settings. In particular, this study employs wireless sensing units (WSUs) to provide continuous and autonomous monitoring in an area where elevated noise levels are a concern due to buildings being located near high traffic areas. The paper is structured, as follows: (1) in Section 2, the state of the art of wireless acoustic sensor networks is discussed; (2) in Section 3, the materials and methods for the proposed wireless sensing unit is described; (3) in Section 4, the results of a field deployment are provided; and, (4) Section 5 concludes the paper with a discussion.

## 2. State of the Art of Wireless Acoustic Sensor Networks

Noise is traditionally measured using handheld devices (e.g., sound level meters) that require manual operation by a trained user, thus resulting in fewer data points over shorter periods of time. As a result, this lower resolution of data acquisition does not capture the full spectrum of noise that a location can experience throughout a day or even longer time period. In order to account for this variability, these few data points are often input into a model that attempts to predict noise levels over extended periods of time [17]. These models, however, tend to be inadequate for today’s dynamic world. To better understand the impact of noise, a higher level granularity is required and the continuous monitoring system that is offered through wireless acoustic sensor networks (WASNs) is ideal.

WSNs have been used in numerous other smart city applications, which can add to the literature when understanding the challenges and capabilities that are associated with this technology. Air quality is a common issue in urban settings and numerous researchers have focused on using WSNs to monitor this variable [18,19]; however, the performance of the transducer has been shown to be a common issue [20]. In [21], it was proposed to use wireless sensors to monitor a less traditional variable of solid waste management for dumpsters in order to optimize fuel and operation time of garbage trucks. In [22], the authors use WSNs for long-term monitoring of storm-water facilities, while simultaneously leveraging cloud services to store data. While these studies provide valuable insight into WSN technology, they do not specifically address the challenges that are presented with WASNs, such as the need for fast sampling rates and balancing data management with on-board computations.

Numerous researchers have focused on developing WSNs for noise monitoring purposes and typically have demonstrated their effectiveness in short term deployments. In [23], a customized noise level meter was designed and tested, which offloaded computations from the sensor node, while simultaneously minimizing overall power consumption across the network. In [24], Hakala et al. used wireless sensors to monitor five minutes of traffic noise, using a self-developed CiNet platform. In [25], the Raspberry PI platform was used as the sensing unit, which leveraged cloud connectivity for data storage. In [26], a wireless acoustic sensor was proposed that utilized Wi-Fi for network communication. These studies highlighted some of the challenges associated with WASNs, such as calibrating the node’s microphone and carefully considering data processing techniques. These were also relatively short term deployments, several minutes to several hours, and they did not consider the overall longevity of the network.

The rapid evolution of the wireless sensing technology has enabled researchers to provide mobility to sensing networks, thus providing measurements that are relevant to all of a human’s daily activities, as opposed to a single location. As with static urban sensing (i.e., permanent location deployment), mobile urban sensing has been applied to numerous smart city applications that use transducers to measure various input variables, with noise pollution being no exception. In [27], a wireless sensing network was installed on urban buses to enable real-time mobile monitoring, while also addressing several challenges, such as establishing optimal locations for noise measurements and general hardware issues. In [28], a hybrid system of static and mobile acoustic sensing was presented, which used smart phones as the mobile infrastructure to enrich the data collection process. While the goal of this study was not to specifically engage the general public in data collection through the use of smart phones, it does cross over into a relatively new area of data acquisition, termed participatory mobile sensing.

Participatory mobile sensing uses smart phones and the general population for data collection through open access repositories. Applications have gained popularity due to the prevalence of consumer smart phones, as well as a general public interest [29]. This form of data collection has been applied to noise monitoring and demonstrated in several open-access platforms, such as City Soundscape [29] and CITI-SENSE [30]. One challenge that has been identified with this form of data collection is the non-uniformity of smart phones, which can result in inconsistent measurements [31,32,33]. Several researchers have proposed implementing algorithms according to a specific phone in order to overcome these limitations [34,35], but this still remains a challenge with the technology.

When extending WASN deployments from short-term to long-term, regardless of whether it is a static or mobile network, additional challenges are encountered, such as data management and network longevity. In [36], an area-based measurement method was proposed that leveraged subsets of the network for data collection, while allowing other subsets to sleep, thus increasing the longevity of the network. In [37], a WASN was demonstrated that integrated both long term and short term monitoring stations, while presenting data in a user-friendly web application. In this paper, the details of power management, overall network maintenance, and data storage were discussed. In [38] the authors propose a novel classifier that is integrated into a long-term WASN in order to identify the noise source. In addition to validating the classifier, the study also discusses power challenges and data management technique. These studies most closely align with the work presented in this paper as they demonstrate long-term monitoring applications of WASNs.

## 3. Materials and Methods

This section contains the different subsections where the design and creation of the prototype, along with the data processing strategies, are explained.

### 3.1. Background: Sound Level

Acoustic waves are pressure fluctuations that transmit through a medium, such as air or water. Sound is perceived by the human ear when these acoustic waves propagate through the auditory system and are converted into equivalent neurological stimuli that are perceived by the auditory cortex. Similarly, a microphone converts these pressures fluctuations into an electric signal that can be processed while using various parameters to quantify the noise. One parameter that is generally used to assess sound exposure to humans is sound pressure level (SPL), which is a quantification of the sound intensity. SPL, or *L_p_*, is calculated as

(1) $$\;L_{p}=20\;\log_{10}\frac{p_{1}}{p_{0}},\;$$

where *p*_0_ is a reference value of 20 μPa, which is considered to be the lowest intensity that can be heard by most humans, and *p*_1_ is the measured sound pressure level of a given sound in units of μPa. As the human ear has a broad range of intensities that it can perceive, SPL is measured in decibels and is represented on a logarithmic scale.

The human ear is also able to hear a wide range of frequencies, from 20 Hz to 20 kHz. However, the ear does not have equal sensitivity across this frequency range, and therefore, in sound measurement, the sound signal is often frequency-weighted so that it matches the ear-perceived level. The IEC (International Electrotechnical Commission) has defined several weighting schemes, termed A, B, C, and D weighting, in IEC-61672-1:2013. In particular, the A-weighting filter emphasizes frequencies from 3 to 6 kHz, to which the human ear is most sensitive, and attenuates very high and very low frequencies, to which the human ear is less sensitive. As such, the A-weighting filter is most commonly used in handheld SPL devices and is most applicable for this application. The A-weighting filter can be described according to the transfer function [39].

(2) $$\;H_{A}\left(s\right)=\frac{7.39705\times 10^{9}\cdot s^{4}}{{\left(s+129.4\right)}^{2}{\left(s+76655\right)}^{2}\left(s+676.7\right)\left(s+4636\right)}\;.\;$$

As noise typically fluctuates significantly, even over short periods of time, the loudness of the noise source is better represented as the root mean square value of the instantaneous sound pressure level Equation (1) over a given time interval, *T*, and it is denoted as *L_eqT_*, such that

(3) $$\;L_{eqT}=10\log\left(\frac{1}{N}\overset{N-1}{\underset{i=0}{\sum }}\frac{p_{i}^{2}}{p_{0}^{2}}\right),\;$$

where *p*_0_ is again the reference sound pressure level, *p_i_* is instantaneous sound pressure level and *N* is the number of samples taken in time *T*. Three time-weighting values have been standardized in IEC-61672-1:2013: ‘*F*’ or Fast (*T* = 125 ms), ‘*S*’ or Slow (*T* = 1000 ms), and ‘*I*’ or Impulse (*T* = 35 ms), and are often offered as different data collection methods on handheld SPL devices.

Thus, standardized methods exist for quantifying noise and typically handheld SPL devices are used for this purpose. These devices, however, require constant oversight by the user for operation and as a result, only offer discrete and limited views of urban noise. Wireless sensor networks that are capable of continuous and autonomous data collection, while still maintaining the precision and capabilities of handheld devices, have the potential to provide more insight into urban noise and future urban planning.

### 3.2. System Prototyping

#### 3.2.1. Hardware Design

**Figure 1:**
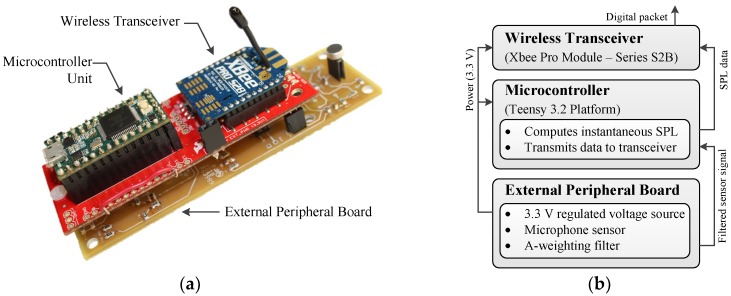
(**a**) Wireless sensing unit prototype with microcontroller unit, wireless transceiver, and the external peripheral board; (**b**) Block diagram depicting function of the complete system.

The wireless sensing unit is comprised of three main hardware components: an external peripheral board, a low-power microcontroller unit (MCU), and a wireless transceiver (Figure 1). While various MCUs can be utilized in noise monitoring applications, the Teensy 3.2 Platform [40] was chosen due to several attractive qualities. This platform has computational capabilities that are provided by the NXP MK20DX256VLH7 microcontroller clocked at 72 MHz with 64 kB of random access memory (RAM). It contains 34 digital I/O pins and 21 analog input pins, allowing it to interface with numerous external peripherals. Embedded code for the platform is also well supported on the Arduino IDE while using the Teensyduino, making it extremely easy to use. When compared with other MCUs, however, it does have higher power consumption (40 mA at 3.3 V in its active mode), and, as a result, the unit is put into sleep mode whenever not in use, thereby reducing its power consumption to 50 μA.

The Teensy 3.2 interfaces with an XBee Pro Module-Series 2SB [41], via the Teensy XBee adapter board, in order to communicate data wirelessly to a centralized repository. It uses the Zigbee protocol that is based on the IEEE 802.15.4 communication standard. The XBee operates in API mode to establish a point-to-multipoint mesh network, thus enabling nodes to communicate to a centralized base unit, as well as peer-to-peer. Additionally, API mode allows for the user to embed additional information into a packet, such as the transmitting WSU identification number, which allows for easier data analysis at the centralized repository. The XBee also has a large power consumption of 205 mA at 3.3 V in transmitting mode, but can be put into hibernation mode whenever not in use, thereby reducing its power consumption to 3.5 μA. Both the Teensy and the XBee are docked on the Teensy adapter board, which has a footprint of 8.7 × 3.4 cm.

**Figure 2:**
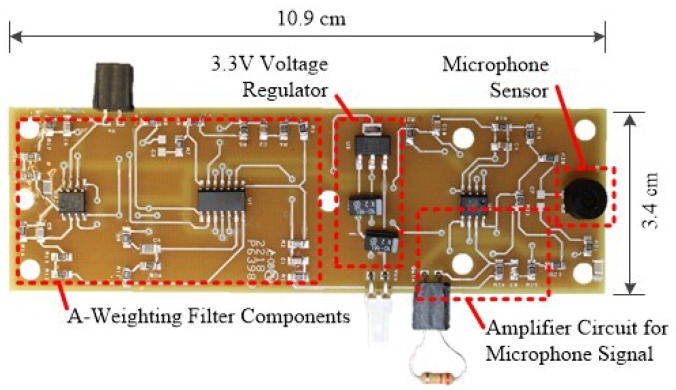
Printed circuit board layout for external peripheral board containing voltage regulator, microphone sensor, and A-weighting filter components.

**Figure 3:**
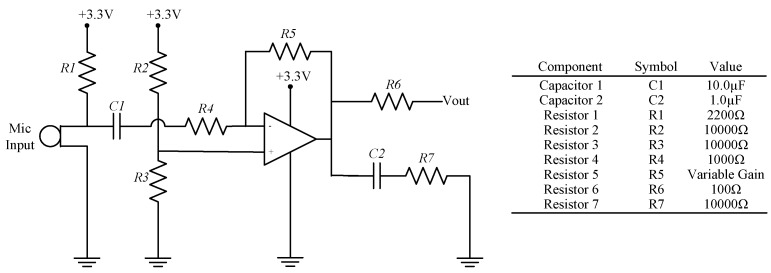
Circuit diagram of inverting amplification circuit for microphone signal.

As this study is focused on urban noise monitoring, the MCU interfaces with an external peripheral board that contains a voltage regulator for the entire WSU, a microphone sensor, and analog circuitry for the A-weighting filter (Figure 2). A POW-1644P-B-R Omni Directional waterproof microphone, a low-cost off-the-shelf component that is manufactured by PUI Audio, is used as the sensor for the acoustic emissions. The microphone provides a high sensitivity (−44 ± 3 dB) operating range from 50 Hz to 20 kHz and a signal-to-noise ratio of 60 dB. As shown in the following section, the microphone was found to have comparative capabilities with the handheld SPL device. In order to fully maximize the capabilities of the microphone, its voltage output is input into an inverting amplifier circuit with a variable gain that can be adjusted with a removable resistor (Figure 3).

**Figure 4:**
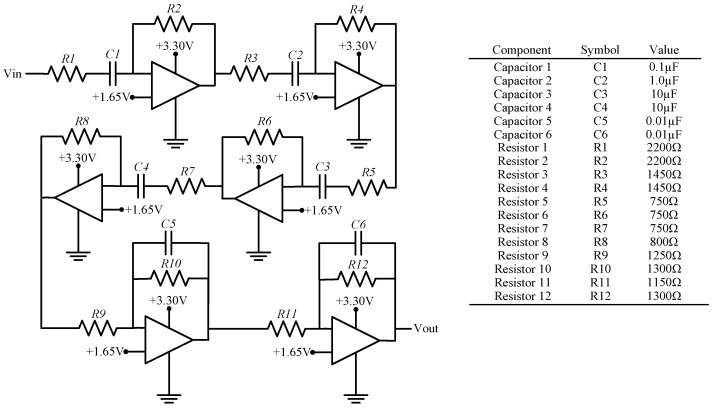
Circuit diagram of the A-weighting filter constructed on the external peripheral board.

**Figure 5:**
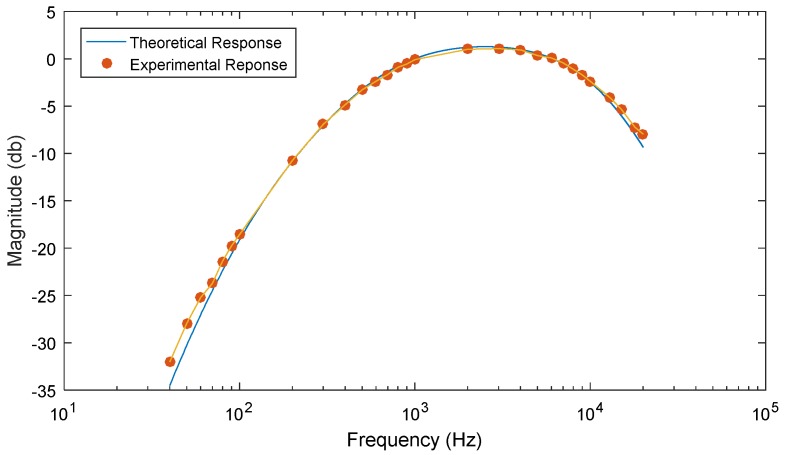
Comparison of the A-weighting filter output from the theoretical transfer function Equation (2) and the experimental circuit board.

Additionally, in order to more effectively emulate the human ear, the A-weighting filter was built in analog circuitry and used to pre-process the microphone signal prior to being sampled by the MCU (Figure 4). To properly capture the characteristics of the filter, four high pass and two low pass active filters were cascaded together, as shown in Figure 4. The gain of the circuit was measured for various input frequencies and was shown to closely match the theoretical response derived from the transfer function provided in Equation (2) (Figure 5). It is important to note that the A-weighting filter could have been implemented as a digital filter as part of the on-board processing strategies of the MCU, however, this would require significantly more computations and it has potential to prohibit real-time data acquisition capabilities. Building the circuit in analog components alleviates this concern. On the other hand, it does make the system less flexible in general, but if a different weighting filter is desired then the external peripheral board can easily be modified. 

The fully assembled wireless sensing unit containing the external microphone peripheral board consumes approximately 245 mA at 3.3 V of power in transmitting mode, 40 mA at 3.3 V of power in computing mode, and 54 μA at 3.3 V of power in sleep mode. It has a small footprint of 3.4 × 10.9 cm, thus allowing for it to be inconspicuously installed in virtually any location, and it costs approximately $75 USD in components (Table 1). As the WSU is comprised primarily of commercial-off-the-shelf components, assembly of the node requires minimal time, with the biggest time being devoted to populating the external peripheral board. When including the estimated assembly costs of $60 USD, the total cost per node is $135 USD.

#### 3.2.2. Software Implementation

**Figure 6:**
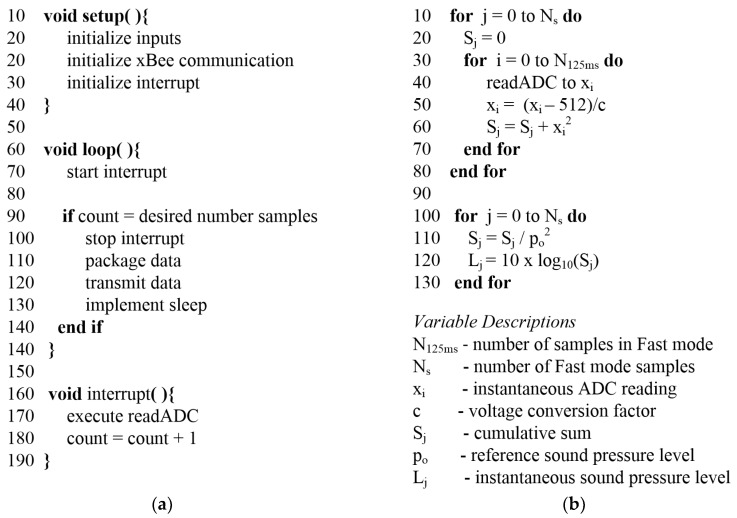
(**a**) Pseudocode for the application architecture on the wireless sensing units (WSU); (**b**) Pseudocode for the computation of the instantaneous sound pressure level embedded on the WSU.

The application layer of the node’s software was written using the Arduino IDE, while interfacing with the Teensyduino add-on. The code leverages several built-in libraries, including the Timer library for interrupts, the XBee API library for communication with the transceiver, and the Snooze library for implementing sleep mode. The pseudocode that is depicted in Figure 6a, provides the basic architecture for data acquisition, communication, and sleeping. As is standard for programming on an Arduino IDE, the initialization is written in the setup function and the control code is written in the loop function. In the loop function, the timer is started and all data acquisition is triggered based on a software timer interrupt. Once the desired amount of data has been collected, then it is packaged into packets using functions that are defined by the XBee API library and wirelessly transmitted. After all the data has been transmitted then the microprocessor enters sleep mode using the Snooze library. After a specified amount of time, the microprocessor wakes and restarts the loop.

The data acquisition and subsequent calculations were carefully considered so as to ensure real-time processing capabilities. As shown in Figure 1a, the microphone signal is pre-processed through several analog circuits before being sampled by the analog-to-digital converter (ADC) on the MCU. These analog circuits modify the signal as needed, thereby reducing the number of required digital computations on the MCU. As the acoustic signals of interest, such as traffic, typically have frequencies below 10 kHz and as the human ear generally attenuates noise at frequencies above 6 kHz, the MCU’s ADC was programmed to sample at 20 kHz for this study. Sampling at this rate has potential to accumulate vast amounts of data which would be prohibitive to transmit wirelessly to a centralized repository for further analysis. As data transmission is the most costly state in terms of energy consumption, minimizing the amount of transmitted data is preferable. As such, the embedded code on the MCU is designed to perform Fast time weighting calculations, as defined in Equation (3). This results in the instantaneous sound pressure level, *L_eqT_*, being calculated every 125 ms using 2500 ADC data points (= 0.125 s × 20,000 samples/1 s).

The pseudocode that is depicted in Figure 6b provides the basic steps for computing *L_eqT_* on the MCU. The algorithm starts by sampling the pre-processed microphone signal using the Teensy’s 10-bit ADC. The value read from the ADC is scaled into a voltage reading, squared, and then added to a cumulative summation term. With the Teensy operating at 72 MHz this takes a total time of approximately 22 μs, which fits within the sampling period of 50 μs, and therefore, can be completed before the ADC acquires the next data point. After 125 ms of data, or 2500 data points, have been acquired, then the next step is to apply the logarithmic function to the cumulative sum term. This operation, however, requires approximately 64 μs of computation time on the Teensy which consumes more time than the allotted 50 μs sample period when sampling at 20 kHz. Thus, in order to not miss any data during the data acquisition period, this final manipulation of the data is applied after the entire data collection period has been completed. It should be noted that the looping mechanism shown in Figure 6b is driven by the interrupt software on the Teensy. As such, this code is actually embedded in Lines 160–190 of Figure 6a. The interrupt stops when the variable count equals the desired number of samples, which is equivalent to N_s_ × N_125ms_. Additionally, the final manipulation of the data (Lines 100–130 in Figure 6b) occurs during the packaging of the data (Line 110 in Figure 6a). 

**Figure 7:**
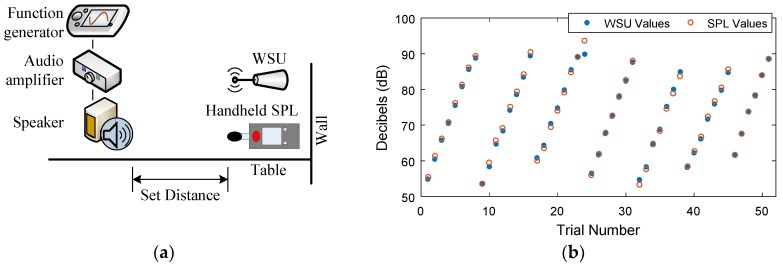
(**a**) Indoor test configuration for comparing the WSU measurements against the handheld SPL device; (**b**) Comparison of WSU measurements to handheld SPL device measurements for tones of varying frequency (Trials 1–8 = 500 Hz, Trials 9–16 = 1000 Hz, Trials 17–24 = 2000 Hz, Trials 25–31 = 3000 Hz, Trials 32–38 = 4000 Hz, Trials 39–45 = 5000 Hz, Trials 46–51 = 6000 Hz) and loudness.

The scaling factor shown in Line 50 of Figure 6b, as well as the resistor value that is shown in Figure 3, are used as calibration tools for computing *L_eqT_*. The Teensy computations were calibrated against the CEL-246 Data Logging Integrating Sound Level Meter [42], which is a high performance Type II sound level meter. It is capable of measuring *L_eqT_* using Fast time weighting, as well as the A-weighting filter. For the calibration test, a 1 kHz sine wave was output from a function generator, passed through an audio amplifier, and played on a professional speaker, while both the handheld SPL device and the WSU recorded the resulting *L_eqT_* in decibels (Figure 7a). The scaling factor and resistor were modified on the WSU in order to fully leverage the range of the Teensy’s ADC and also to better match the handheld SPL device’s calculations of *L_eqT_*. This resulted in a resistor value of 330 kΩ and a scaling factor of 275.0.

The accuracy of the WSU was tested while using the CEL-246 handheld SPL device for the test configuration shown in Figure 7a. Both the handheld meter and the WSU were placed 25.4 cm from a function generator that output sinusoidal waveforms of varying frequencies (ranging from 500 Hz to 6000 Hz), to an audio amplifier and then a speaker. The amplitude of the generated signal was increased to achieve varying loudness, ranging from approximately 55 dB to 90 dB in 5 dB increments. Due to limitations of the output range of the function generator, some loudness values could not be achieved at certain frequencies. During each trial, the *L_eqT_* was recorded by both devices for 60 s, while using the Fast time weighting and the A-weighting filter. Due to the limitations of its recording capabilities, the handheld SPL device logged one data point every one second with a resolution of 0.1 dB. The WSU, on the other hand, logged one data point every 125 ms to be consistent with the Fast time weighting and with a resolution of 0.01 dB. Both devices produced consistent measurements during the 60 s trials, with all the trials producing average standard deviations of 0.047 dB and 0.089 dB for the handheld SPL device and the WSU, respectively. As can be seen from Figure 7b, the WSU and the handheld SPL device produce similar results, with the exception of one measurement in which the averaged WSU measurement was 3.90 dB below the averaged SPL measurement (frequency = 2000 Hz, loudness = maximum). As all other tests produced significantly smaller differences, less than ±1.5 dB difference, this one trial can be considered to be an outlier in which errors in measurement techniques may have impacted the result. As such, this test indicates that the WSU is an appropriate substitution for the SPL device. 

#### 3.2.3. Centralized Repository

**Figure 8:**
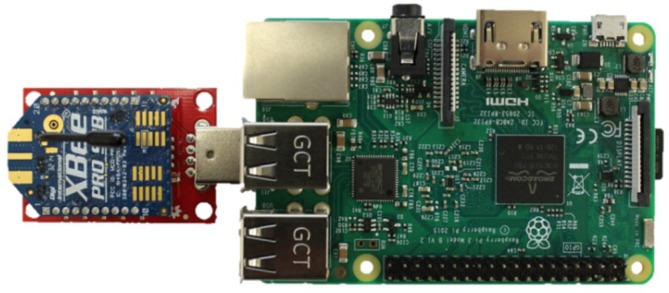
Raspberry Pi with attached XBee coordinator.

The low-cost and low-power features of the WSUs allow for a dense network to be deployed, which results in a higher resolution of information to be collected. Each unit is capable of communicating directly to a centralized node, as well as in a peer-to-peer fashion which enables multi-hop communication schemes if necessary. To facilitate the network, the Raspberry Pi 3 (RPi) single board computer (Figure 8) is deployed as the centralized node that collects and stores all information from all the other nodes in the network. The RPi uses the Broadcom BCM2387 system on a chip and contains 1 GB of RAM, as well as on-board 802.11n Wi-Fi capabilities [43]. The RPi interfaces with an XBee coordinator via the RPi’s USB port, while using the built-in serial library, which enables communication with the WSUs in the network. This allows the RPi to collect the processed data from the network of WSUs and store it on-board as a text file. When a packet is received from a WSU, the RPi logs a timestamp and the packet of information into the text file, using the built-in binascii and datetime libraries. This allows for the WSU ID number and the packet payload that contains the processed *L_eqT_* data to be extracted at a later time. While using Secure Shell (SSH) or Virtual Network Computing (VNC), the RPi can be queried remotely to access and download the received data.

#### 3.2.4. Data Acquisition and Transmission

**Figure 9:**
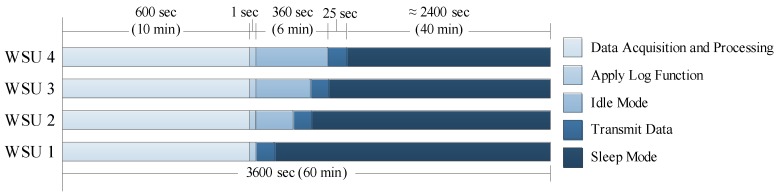
Hourly task scheduling for WSU.

In order to understand the long-term dynamics of urban acoustic emissions, the WSUs were programmed to collect data every hour for 10 min (or 600 s) (Figure 9). During this period of data acquisition, each WSU executes the inner loop of calculations (Lines 30–70) that are outlined in Figure 6b. When sampling at 20 kHz, this results in 2500 data points being averaged over a 125 ms period, or a total of 4800 (= 600 s/0.125 s) processed data points being stored locally. After the 10 min data acquisition period, the logarithmic operation is applied to each data point, consuming approximately 1 s of computation time for all 4800 data points. This processed data is then transmitted to the centralized node, with 50 data points in each packet payload. 

To better facilitate the data transmission and prevent packet collision and data loss, the WSUs are hardwired to delay data transmission based on their unit ID number. Wireless transmission of the processed data requires approximately 25 s. Immediately after processing all of the data, WSU 1 transmits its data to the centralized repository and then enters sleep mode. WSU 2 waits 120 s, transmits its data, and then enters sleep mode. This continues with each unit waiting an allotted amount of time before transmitting data and then entering sleep mode. Prior to entering sleep mode, each node determines the amount of time that has passed since awaking from the previous sleep mode, which dictates how long to sleep until the next data acquisition period. The timing of a typical data acquisition period and sleep is depicted in Figure 9. Each WSU consumes 810 mW of power in active mode, 132 mW of power in transmitting mode, and 180 W of power in sleep mode (at 3.3 V). When oscillating between these three states over a one hour period, the WSU is able to operate continuously for seven days without requiring human intervention or a new power source (i.e., batteries).

## 4. Results

**Figure 10:**
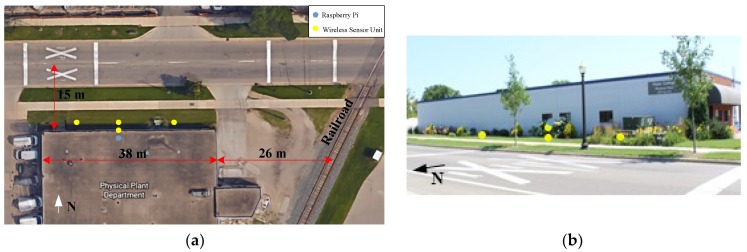
Layout for field deployment of network of WSUs: (**a**) plan view; (**b**) elevation view.

A network of WSUs was deployed for long-term noise monitoring in Holland, Michigan, USA on the campus of Hope College (Figure 10). This area is of particular interest as it is a part of campus that is close to a highly used railroad and a relatively busy road, making noise a concern in buildings. Four WSUs and one centralized repository node were installed in the network. WSU 1 was placed on the ground 6.5 m from the Northwest corner of the Physical Plant building, WSU 2 was placed near the center of the building, approximately one-meter off the ground, WSU 3 was placed near the center of the building on the ground, and WSU 4 was placed on the ground 12 m from the Northeast corner of the building. The Raspberry Pi was installed inside the building, near Units 2 and 3.

**Figure 11:**
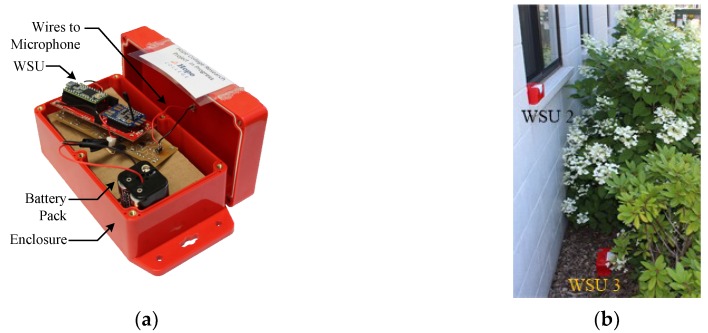
(**a**) Packaging of WSU for field deployment; (**b**) Field deployment of WSUs.

Each WSU is packaged in a SparkFun Electronics waterproof plastic container (dimensions 15.8 × 9.0 × 7.4 cm, cost: $10 USD) that is designed to protect the wireless sensor electronics from the outdoor environment (Figure 11). All components of the WSU, including the battery pack, are packaged in the container, with the exception of the microphone. The microphone is instead connected to the external peripheral board through leads and is mounted externally to the container, so as to not inhibit the sensor’s ability to detect acoustic emissions. The wires used to interface the microphone to the WSU are passed into the enclosure through drilled holes, which are sealed with epoxy to ensure that the enclosure remains waterproof. Inside the container, the WSU and battery pack are attached while using epoxy to bottom surface of the enclosure. The RPi is packaged in a standard case from the Raspberry Pi foundation, thus protecting it while allowing for easy access if needed.

**Figure 12:**
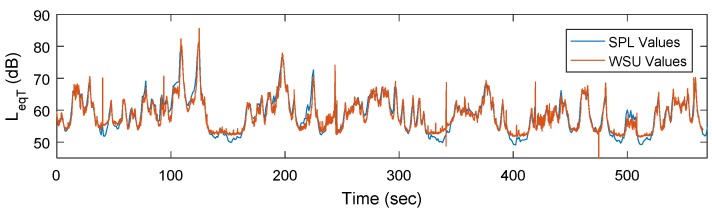
Field measurement comparison between handheld sound pressure level (SPL) device and WSU.

Prior to the full field deployment, a ten-minute test was conducted while using a single WSU and the handheld SPL meter, in order to verify the calibration of the WSU in an outdoor setting, as well as for noise sources at long-range distances. Figure 12 shows the calculated *L_eqT_* values using both measurement devices. Similar to the test set-up in Section 3.2.2, one data point was logged every 1 s for the handheld SPL device and every 125 ms for the WSU. As a result, the WSU is occasionally able to detect larger peaks than the handheld SPL device. On the other hand, the lower limit of the WSU’s measurement range is approximately 50 dB and as a result, it cannot capture the lower end of the noise spectrum as depicted at a time of approximately 150 s in Figure 12. While this is a limitation of the system, this does not significantly detract from the WSU’s capabilities of detecting trends, as well as significant noise events, such as trains or heavy traffic. All the units in the network were tested in this manner and they showed similar results.

**Figure 13:**
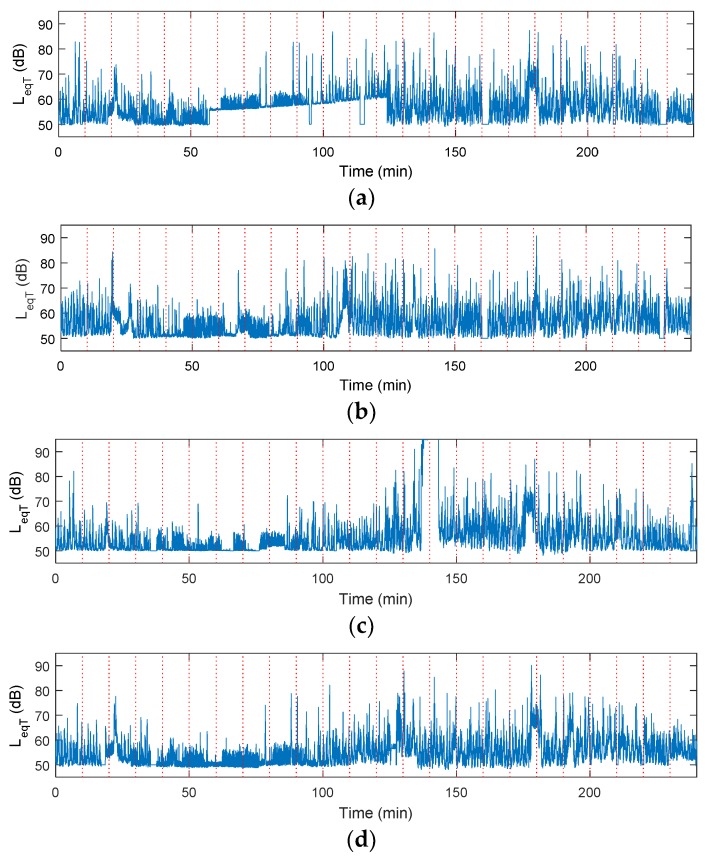
Sample noise data from 24 h collection period for (**a**) WSU 1; (**b**) WSU 2; (**c**) WSU 3; (**d**) WSU 4; dashed vertical lines represent a 10 min collection period that occurs once per hour.

The network was deployed to allow for continuous and automated data collection and it operated for two weeks. Data was collected by the WSUs, as described in Section 3.2.4 and stored by the centralized repository, as described in Section 3.2.3. It was retrieved from the repository using VNC and reformatted using MATLAB. One 24 h period of data collection is shown in Figure 13, with Time 0 representing 00:00 hours. In general, the units are constrained to measuring 50 dB on the lower end and record values as high as 90 dB. 

**Figure 14:**
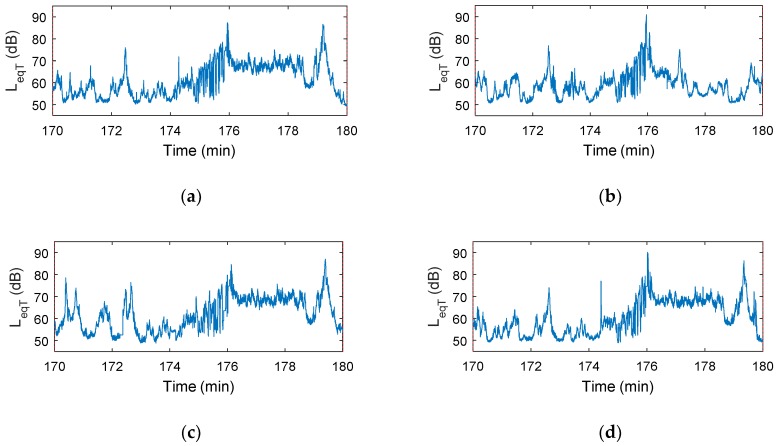
Sample noise data from a 10 min collection period for (**a**) WSU 1; (**b**) WSU 2; (**c**) WSU 3; (**d**) WSU 4.

Additionally, as all the units are located within 20 m of each other and are collinear, they follow similar trends. One data collection period, extracted from the 17th hour, is shown in Figure 14, in which all units detected a loud noise that occurred for a long duration, potentially a train. In this case, Unit 2 depicts a slightly different profile than the other three nodes which is due to its location along the building. This node is mounted on a window sill that is slightly inset into the building. As the train runs perpendicular to this side of the building some noise may be blocked from the sensing unit. The other three units are located on the ground along the length of the building and should experience similar sound exposure. During this 10 min trial, the units detect peak values that are close to 90 dB, which is expected in urban areas, but are highly undesirable for humans [11]. While it is only one peak value, the nose level does remain above 70 dB for almost 2 min. 

**Figure 15:**
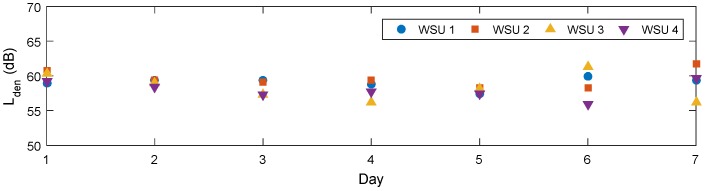
Day-evening-night sound pressure levels over week period.

The average day-evening-night sound pressure level, *L_den_*, is another commonly used noise indicator and provides a weighted average over the entire day by adding a 5 dB penalty for noise occurring in the evening and a 10 dB penalty for night noise [12]. Figure 15 shows this indicator for a seven-day period for the four WSUs. In general, *L_den_* was approximately 60 dB for all of the trials. This value may be slightly elevated as WSU cannot measure below 50 dB; however, when considering the time history plots (Figure 15), a slight decrease in noise levels can be observed during the night hours, but there is still a fairly consistent flow of traffic and freight trains continue to run through the area throughout the night. 

## 5. Discussion

In this work, the details of the design of a wireless sensing unit that is capable of data acquisition of acoustic noise and its deployment in a long-term field validation is presented. Through the hardware and software validation, presented in Section 3, it was shown that the WSU can be used as an appropriate substitution for the traditional methods of noise measurement. The on-board computational capacity of the unit is leveraged to calculate equivalent sound levels and reduce the amount of data transmission, which results in significant energy savings at the node. Additionally, the calculations are carefully structured so that the node does not miss any data during its acquisition phase. 

In Section 4, the long-term monitoring of a network of four WSUs was described in detail. In general, the units were shown to be able to capture significant noise events and monitor over long periods of time. At this specific location of interest, peak values reach up to levels of 90 dB and the 24 h noise indicator was calculated as approximately 60 dB, which can cause long-term health defects, such as high blood pressure [10]. From this brief test alone, it can be recommended that steps be taken to reduce exposure to this noise for the inhabitants of nearby buildings.

Through this deployment, several areas of future work were highlighted. First, the dependence of the WSUs on finite power supplies (e.g., batteries) is a challenge, as it prohibits the longevity of the network and prevents full autonomy by requiring periodic maintenance by the user. When the batteries begin to deplete, as is occurring in Figure 13a between time 60 and 120 s, the voltage input to the WSU’s ADC increases, which results in incorrect reporting of *L_eqT_*. Numerous other studies have shown that replacing these batteries with alternative energy sources, such as solar power [38], powering units off of the grid [37], or using city light systems, as is implemented in Chicago’s AoT, can easily increase the overall longevity of the network. 

Additionally, as currently implemented, the centralized repository does not query a WSU again if it does not receive a packet and approximately 2.6% of the data is currently being lost during transmission. As a data point is collected every 0.125 s, this only represents 15.6 s of data combined across all units in a 24 h period which is not extremely significant when considering the purposes of this data. However, in the future, the handshaking capabilities of the XBee transciever will be employed to prevent data loss in the network. Alternatively, other studies that use WSNs for Smart City applications have explored using alternative wireless communication methods with varying success and such options will also be considered in the future [44]. Finally, once the data is acquired, it currently is deposited in a repository and no significant data analysis occurs. Future work will include implementing data mining techniques, as well as developing methods for further analyzing the data. 

The WSU developed in this study proved to be an appropriate replacement for traditional handheld SPL devices in that it possesses the same computational output of the instantaneous sound pressure level, but it is also capable of continuous and autonomous data acquisition. A fully operational prototype was demonstrated in a long-term multi-unit deployment. Through this deployment it was demonstrated that the overall trends in urban noise can be observed and better understood, thus allowing for urban planners and engineers to improve city designs so as to accommodate the rapid urban growth and maintain the overall quality of life.

## Figures and Tables

**Table 1:** Wireless sensing unit cost breakdown.

Part	Commercial Name	Price (USD)
Microcomputer	Teensy 3.2 Platform	$20
Wireless transceiver	XBee Pro Module—Series 2SB	$20
Adapter board	Teensy 3.1 XBee Adapter	$10
Microphone	PUI Audio Microphone	$5
Ext. Peripheral Board and Components	-	$20
Assembly Cost	3 h @ $20/h	$60
Total Price per Node		$135
